# Predictors, Mediators and Moderators of Police Work-Related Stress: A Scoping Review

**DOI:** 10.3390/ijerph20032253

**Published:** 2023-01-27

**Authors:** Yuen-Kiu Cheung, Jessica Chi-Mei Li

**Affiliations:** Department of Applied Social Sciences, The Hong Kong Polytechnic University, Hong Kong, China

**Keywords:** police, work-related stress, stressors

## Abstract

Owing to the complication in organisation, the dangerous job nature and the rise of demonstrations and protests across the world in the past decade, police work-related stress has become a topic of global concern. This review aimed to provide an understanding of predictors, mediators and moderators of police work-related stress from a multi-level perspective. Using a scoping review approach underpinned by the six-stage methodological framework, studies were found from six electronic databases (MEDLINE, Web of Science, Sociological Abstracts, Scopus, PsycINFO and PsychiatryOnline) and grey literature sources. Thirty studies were yielded across 35,446 participants from 12 locations. This review contributes to a systematic understanding of the factors affecting police work-related stress by identifying six predictors, four mediators and three moderators. It then discusses limitations and future research.

## 1. Introduction

### 1.1. Police Work-Related Stress

The nature of police work can be dangerous and stressful from exposure to critical incidents and traumatic duties/events, such as dealing with abused children and dead bodies, pursuing suspects, and the threat of injury or death and violence [1,2,3,4,5,6,7]. Other than the job nature, the recent conflictual socio-political environment in many cities across Asia, Europe and America has been likely to put an additional burden on police officers. For instance, in 2019, the anti-extradition law indicated the beginning of social unrest in Hong Kong, and strikes and protests began in France due to the anti-pension system reform. In 2020, protests against racism and police brutality spread in the United States (US) following the death of a black man. As a result of all these protests and riots, increasing work demand and tension in the police–public relationship actually created adverse working conditions for police officers. Hence, police work-related stress is a timely topic of investigation. In this review, the terms work-related stress, job stress, occupational stress or workplace stress were used interchangeably. “Work-related stress is the response people may have when presented with work demands and pressures that are not matched to their knowledge and abilities and which challenge their ability to cope” [8]. One outcome of chronic work-related stress is burnout [9], defined as “an acute stress disorder or reaction characterised by exhaustion resulting from overwork, with anxiety, fatigue, insomnia, depression, and impairment in work performance” [10] (p. 106). Signs of burnout include emotional exhaustion, depersonalisation, reduced personal accomplishment, being frenetic, underchallenged or worn-out, and experiencing cynicism and lower professional efficacy [11,12,13]. For formulating feasible policies and practice to reduce work-related stress and burnout, identifying the sources of work-related stress is of great importance not only for the individual well-being of police officers but also for the good of police organisations and the safety of the general public. Conducting a scoping review of the sources of police work-related stress is a fundamental step.

### 1.2. Previous Reviews

Over the past few years, there have been several publications synthesising and analysing the sources of police work-related stress with the systematic review method. For example, a systematic review of the relevant publications from 1990 to 2017 was completed [14]. Based on authors’ analysis of fifteen selected studies conducted with a cross-sectional design spanning across four continents, they concluded significant associations between organisational stressors and different mental health outcomes among police officers. One systematic review of sixteen articles published from 2006 to 2018 on nine countries examined sociodemographic factors, other individual difference factors, organisational factors and critical incident related factors for anxiety and depression among police officers [15]. Another systematic review of 29 cross-sectional studies conducted across fourteen countries published between 1985 and 2018 summarised risk factors for stress into the following five categories: demographic characteristics, job characteristics, lifestyle factors, negative coping strategies, and negative personality traits [16]. All these recent reviews of the sources of police work-related stress have offered a solid foundation for police stress studies. Despite the contributions, these previous reviews are subject to several constraints. Firstly, the available literature seems to narrowly focus on individual and organisational stressors and scarcely examined other stressors from family and community. Secondly, although the literature has largely uncovered the correlates of police work-related stress, whether the correlates are predictor, mediator or moderator remains unclear. Thirdly, many studies were conducted in the Western context, and understanding of the studies that were conducted under a non-western context remains inadequate. Lastly, a majority of the existing literature used quantitative methodology and research scarcely investigated this topic using qualitative methods. To fill these gaps, the current review is intended to examine the factors affecting police stress directly or indirectly from a multi-level perspective through a scoping review of both quantitative and qualitative studies published between 2011 and 2020.

### 1.3. Current Review and Theoretical Foundation

The sources of police work-related stress come from two domains: organisational (strains related to the agency and management) and operational (strains related to the job nature). Among the explanatory theories of work-related stress and burnout, the job demands–resources (JDR) model [17] and the job demand–control (JDC) model [18] are adopted in this review because the clear theoretical concepts of these two models help elaborate how work stress develops in organisational and operational domains. The JDR model assumes that high job demands cause more exhaustion while a lack of job resources can help enhance engagement. The JDC model posits that workers can reduce job strain through gaining greater job control. Although both models offer a solid theoretical framework for understanding police officers’ work-related stress resulting from organisational factors and job nature, stressors at other levels or domains, such as family and community, are less mentioned. Thus, a multi-level perspective is needed to assist our analysis in this review. Therefore, the key concepts of the JDR model, the JDC model, and the ecological model of occupational stress [19] are adopted in a supplementary manner to provide a theoretical basis for this review. This model asserts that an understanding of occupational stress and stressors requires looking at microsystem (e.g., job content, person–work environment fit, and individual’s family responsibilities), organisational system (e.g., demand of job and leadership), peri-organisational system (e.g., community’s perception of organisation’s service), extra-organisational system (e.g., prevalent attitudes/biases), and the possible moderators (e.g., rank, marital status, years of service, and individual coping strategies). In short, the potential contributions of this review are three-fold. First, it is one of the first scoping reviews of police work-related stress not only looking at factors at different levels but also their interactions through mediation and moderation. Second, regional difference in the demographic factors were delineated and presented clearly in this review. Third, the results of the review may shed light on the possible actions to be taken at different levels to reduce police work-related stress.

## 2. Methods

This scoping review was conducted according to the six-stage methodological framework, including identifying the research question(s); identifying relevant studies and study selection; charting the data and collating, summarising and reporting the results; and consultation exercise [20].

### 2.1. Literature Search

Considering that this review study looks into police officers’ work-related stress from multiple perspectives, it consulted six electronic databases across disciplines (MEDLINE, Web of Science, Sociological Abstracts, Scopus, PsycINFO and PsychiatryOnline) and grey literature sources. To capture the relevant articles, three groups of search terms were chosen. The first group of search terms included ‘stress’, ‘strain’, ‘pressure’, ‘tension’ and ‘burnout’. The second group of search terms included ‘predictor’, ‘antecedent’, ‘cause’, ‘contributing factor’, ‘stressor’, ‘risk factor’, ‘source’ and ‘origin’. The third group of search terms included ‘police’, ‘policeman’, ‘policewoman’ and ‘police officer’.

### 2.2. Literature Selection and Data Extraction

Selection of articles was subject to a number of inclusion and exclusion criteria. Articles were included if they were:

1. Published between 2011 and 2020 (the time period is limited from 2011 to 2020 to yield the most current results and reflect the rise of demonstrations and protests in this decade)
2. Written in English
3. Including police officers
4. Empirical papers

Meanwhile, irrelevant articles were excluded if they were:

1. Published before the year 2011
2. Non-English
3. Including employees from non-policing occupations
4. Book reviews, conference reports, dissertation and editorials

**Figure 1:**
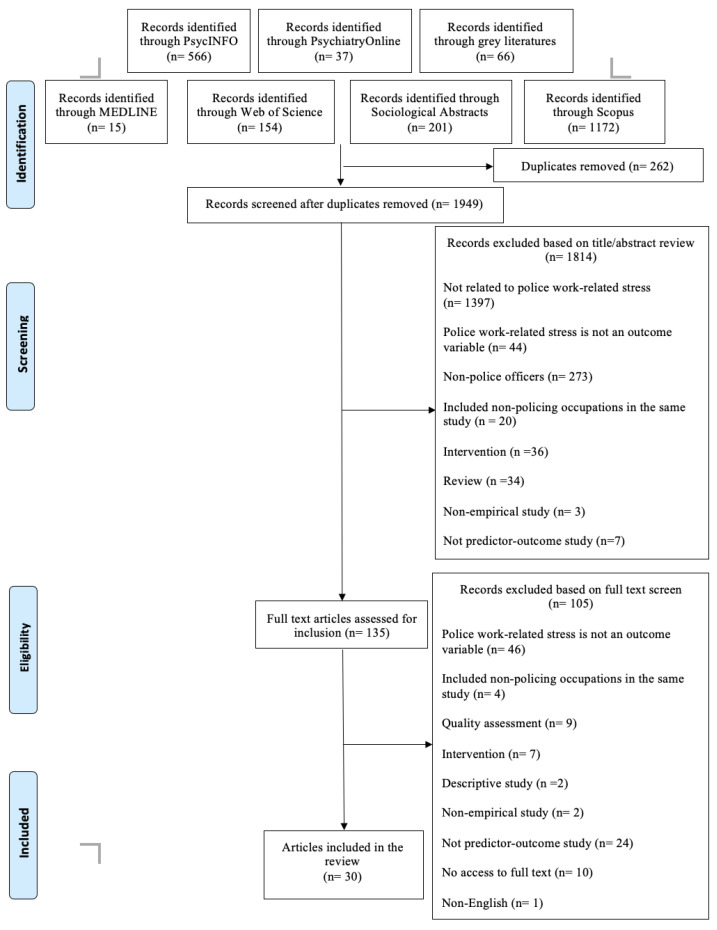
Flow diagram of the search process.

This review found a total of 2211 articles. After removing the duplicated articles, 1949 articles were kept. After excluding the irrelevant articles, 135 articles were identified. The full text version of the remaining articles was scrutinised. By scrutinising the full text articles, 105 articles were excluded for the following reasons: police work-related stress is not an outcome variable; included non-policing occupations in the same study; intervention, descriptive study, non-empirical study and not predictor–outcome study; no access to full text; non-English and cannot pass the quality assessment. Finally, 30 articles were included for the current review. Figure 1 shows the flow diagram of the search process, which is in accordance with the Preferred Reporting Items for Systematic Reviews and Meta-Analysis (PRISMA) guidelines [21].

### 2.3. Quality Assessment

After scrutinising the full text articles, 39 articles were selected and evaluated using standardised critical appraisal tools. They include quantitative studies (*n* = 37), the qualitative study (*n* = 1) and the mixed-method study (*n* = 1). The checklists offered by the Joanna Briggs Institute [22,23] and the Evaluation Tool for ‘Mixed Methods’ Study Designs developed by Long were adopted for quality assessment [24]. The quantitative studies and the qualitative study were reviewed with a set of questions from a checklist and the rating options of ‘yes’, ‘no’, ‘unclear’ or ‘not applicable’. The mixed-method study was reviewed by answering 52 questions from seven review areas (including study evaluative overview, study and context, ethics, group comparability, qualitative data collection and analysis, policy and practice implications and other comments). The first author conducted the review, whilst the second author checked and confirmed the rating by the first author. Both authors had several rounds of discussion to clarify the assessment results. As a result, nine selected articles did not meet the criteria described in the critical appraisal tools. Therefore, 30 articles were included. Table 1 and Table 2 show the assessment result of the quality of selected articles.

## 3. Results

### 3.1. Characteristics of the Included Studies

This review yielded 30 articles across 35,446 participants from 12 locations, including the US [27,28,30,31,34,35,36,39,41,49,53,55,60,62], the UK [46], Spain [37], Greece [38], Poland [58], Sweden [33,45], Finland [52], Turkey [43], Sri Lanka [25], India [40,42,44,48,57], South Korea [29] and China [59] (see Table S1. Summary of study characteristics and main findings). A majority (*n* = 14) of the reviewed studies were conducted in the US. Among the 30 included studies, 29 included studies used a quantitative design, among which 23 used questionnaires/surveys and six used secondary data [30,31,34,35,41,58]. Among the 29 quantitative studies, 20 were a cross-sectional design. One included study used a qualitative design and in-depth interviews [62]. Thus, a majority of the reviewed studies were based on cross-sectional survey data.

The sample of police officers in the selected studies varied in terms of age range (from 18 to 80), gender (53% to 100% for males; 0% to 56% for females), education received (ranging from high school to postgraduate programmes), marital status (44.6% to 83% for married; 0.45% to 55.4% for unmarried), number of children (ranging from zero to seven), race (50% to 87% for White; 0.6% to 55% for non-White), rank (varied from gazetted officers, non-gazetted officers, police officers, sergeants, lieutenants, captains, officer trainees, agents, detectives, constables, corporals or below, supervisors, line officers, sub-inspectors, head constables, inspectors and other positions), tenure (ranging from zero to 44 years), and job nature (varied from patrol function, non-patrol function, daily crime investigation, demanding crime investigation, public order and security, and working in an office) (see Table S1). Notably, this review covers police populations with a diverse background, but more with male, White and US based respondents. Moreover, not all the aforementioned demographic data were collected in the selected studies, and not all the collected demographic data were analysed for their associations with work-related stress.

### 3.2. Demographic Predictors

Table 3 shows the demographic predictors of police work-related stress. The variation of the results regarding demographic predictors of police work-related stress was observed across three regions, namely US, European countries and Asian countries.

#### 3.2.1. US

In this review, among those studies conducted in the US, results relating to the prediction of age were mixed. In some studies, age was a significant demographic predictor [34,35,53], with younger police officers suffering from a higher level of job stress and burnout [34,35,53]. However, others found that age did not predict job stress or burnout [28,39,41]. Regarding the predictive power of gender, mixed results were found. One study highlighted that male police officers had a higher level of job stress [53]. Some studies pointed out that female police officers suffered from an increased risk of work-related stress and burnout [30,60]. However, others found that gender was a non-significant demographic predictor [28,31,34,35,36,39,41]. Results regarding the predictive power of race were mixed, with White police officers suffering from a higher level of job stress and burnout [31,39], but one highlighted that non-White police officers had a higher level of occupational stress [36]. Some pointed out that those belonging to a racial minority experienced less burnout in policing contexts [27,49], whereas others found that race was a non-significant demographic predictor [28,30,34,35,41]. With respect to the prediction of rank, mixed results were found. Higher rank police officers experiencing a higher level of job stress [41], but some pointed out that lower rank police officers experienced more burnout [34,35]. Others found that rank did not predict police occupational stress nor burnout [36,39]. Finally, concerning tenure, although some pointed out that police officers with longer tenure suffered from a higher level of job stress and burnout [31,34,53], others highlighted that police officers with shorter tenure reported a higher level of occupational stress and a greater feeling of emotional exhaustion [36,49]. Several found that tenure was a non-significant demographic predictor [30,35,41,60]. Notably, across 70 municipal police departments and 19 full-service county Sheriff’s offices, although age, gender, race and rank were significant demographic predictors of emotional exhaustion and depersonalisation, these demographic predictors explained very little variation [27].

#### 3.2.2. European Countries

Regarding the influences of demographic characteristics on police work-related stress, variations were found among the countries in Europe. Age was the only significant demographic predictor of job burnout for Polish police officers [58], with older police officers having a higher level of job burnout. However, age was a non-significant demographic predictor of burnout among police personnel working in Sweden [33]. For Finnish police officers, tenure and function were significant demographic predictors of burnout [52], with police officers who worked for less than two years, two to five years or six to 15 years experienced less burnout. Finnish police officers who worked in daily or demanding criminal investigations experienced more burnout, but not those working in public order and security. In the same study, a gender impact was not observed.

#### 3.2.3. Asian Countries

With respect to the demographics impacts on police work-related stress, discrepancies were found among Asian countries. In a police burnout study conducted in Sri Lanka, age and marital status were significant demographic predictors of burnout subtypes [25]. Younger or unmarried police officers perceived a greater feeling of being underchallenged. Unmarried police officers perceived an elevated feeling of being worn-out. This study did not find gender, education, family status, rank and tenure to be significant demographic predictors of burnout subtypes. An Indian police stress study found that education and rank were significant demographic predictors of work stress [42]. Police officers with lower education or holding the rank of constable suffered from an increased risk of work stress. In the same study, the impact of age, gender and tenure were not observed, whereas other Indian police-based stress studies found that age, gender, education, marital status, rank and tenure were non-significant demographic predictors of job stress and burnout subtypes [40,44,48]. A South Korean police burnout study did not find age, education, marital status, rank or tenure to be significant demographic predictors of burnout [29].

### 3.3. Personal Predictors

Several of the reviewed studies found that personal predictors predicted work-related stress and burnout at a significant level [31,33,34,38,49,60], while other studies exhibited mixed results [37,45,46,53]. Among the studies conducted in the US, one found job stress was driven by in/out group status [31]. Police officers who perceived themselves as not a part of the subculture had a higher level of job stress than those who perceived themselves as a part of it. Additionally, individual unfairness, work–life imbalance and more work–life conflict contributed to more burnout [34,49,60]. More positive affectivity was aligned to less burnout but not job stress [53].

Regarding studies conducted in some European countries, among police officers working in the UK, fewer reliability-based personal trust beliefs in the police emerged as a contributing factor, but honesty and emotional-based personal trust beliefs in the police did not lead to an increased risk of workplace stress [46]. As for the Spanish National police members, optimism but not self-efficacy protected against job stress [37]. In a study conducted on Greek police officers, those who had decreased sleeping hours per day or days of physical exercise per week were prone to a higher level of occupational stress [38]. Another study conducted in Sweden indicated that higher stress of conscience led to greater feelings of emotional exhaustion and depersonalisation for both male and female police personnel [33]. Similarly, a higher stress of conscience led to a greater feeling of emotional exhaustion for both genders but only led to an elevated feeling of depersonalisation for male patrolling police officers [45].

### 3.4. Organisational Predictors

One of the sources of work-related stress is from organisation. With reference to a number of related theories, organisational predictors in this review included job demand, job control, job resources, job/workplace satisfaction, organisational commitment, internal procedural justice, and organisational problems.

#### 3.4.1. Job Demand

Results relating to the prediction of job demand were mixed. Excessive workload was a significant predictor of greater feelings of emotional exhaustion and depersonalisation among American law enforcement officers [27], heavy job demands were a significant predictor of a high level of job burnout for Polish police officers [58], and heavy work demand predicted a greater feeling of emotional exhaustion, but not depersonalisation, among Swedish police officers [33,45]. In Asia, a study conducted in Sri Lanka indicated that long working hours, namely 50 h or more per week, were a significant predictor of a greater feeling of being frenetic but not being underchallenged nor worn-out [25]. An Indian police stress study found that heavier job demands, reflected by role conflict, role ambiguity and role overload, were significant predictors of an increased risk of work stress [42]. Another Indian police burnout study found that role ambiguity predicted a greater feeling of depersonalisation and a reduced personal accomplishment but did not predict emotional exhaustion [57]. Work overload predicted an elevated feeling of emotional exhaustion, but did not predict depersonalisation and personal accomplishment [57].

#### 3.4.2. Job Control

Some of the reviewed studies found that job control predicted burnout at a significant level [27,58]. For example, lower control was a significant but weak predictor of greater feelings of emotional exhaustion and depersonalisation among American law enforcement officers [27] and lower job control was a significant predictor of a higher level of job burnout for Polish police officers [58]. A few studies exhibited mixed results [33,45], with lower decision latitude predicting greater feelings of emotional exhaustion and depersonalisation for males and females, respectively, among Swedish police personnel [33,45].

#### 3.4.3. Job Resources

Regarding the predictive power of job resources, mixed results were found. Among the studies conducted in the US, unsupportive supervisors were identified as a source of workplace stress [62]. Less counselling support and social support from co-workers and supervisors were significant predictors of a higher level of job stress and more burnout [41,60]. Regarding the European countries, lower social support was a significant predictor of a higher level of job burnout among police officers working in Poland [58]. Lower social support only predicted a greater feeling of emotional exhaustion for Swedish female police personnel [45]. With respect to the countries in Asia, a study conducted in Sri Lanka indicated that infrequent superior guidance and dissatisfactory higher rank officers’ support were significant predictors of elevated feelings of being underchallenged and worn-out, but not frenetic [25]. An Indian police stress study found that fewer job resources, reflected by input into decision making, formalisation and organisational support, were significant predictors of an increased risk of work stress [48]. Another Indian police burnout study found that lower work support predicted more burnout subtypes [57].

#### 3.4.4. Job/Workplace Satisfaction

Results regarding the predictive power of job/workplace satisfaction were mixed. One study conducted on American law enforcement officers found lower job satisfaction was a significant but weak predictor of a greater feeling of emotional exhaustion [27]. Another study conducted in the US found that dissatisfaction with peers and supervisors predicted greater feelings of emotional exhaustion and depersonalisation, but no depersonalisation for males [49]. Among police members working in Turkey, lower job satisfaction was a significant predictor of an increased risk of work-related burnout [43]. In a study conducted in Sri Lanka, perceived dissatisfaction about the staff adequacy was a significant predictor of greater feelings of being frenetic and worn-out [25]. Perceived dissatisfaction about the infrastructure facilities and dissatisfaction about the allowances and public service were significant predictors of an elevated feeling of being underchallenged. Nevertheless, satisfactory welfare facilities and satisfactory salary did not predict burnout subtypes. An Indian police burnout study found that lower job satisfaction was a significant predictor of elevated feelings of emotional exhaustion and depersonalisation, but higher job satisfaction predicted a reduced sense of personal accomplishment [40].

#### 3.4.5. Organisational Commitment

Another important job resource taken into consideration is organisation commitment. Organisational commitment refers to the degree of an employee’s identification with and involvement in an organisation [63]. In some Asian countries, an Indian police burnout study found that higher continuance commitment predicted greater feelings of emotional exhaustion and depersonalisation. However, its prediction on personal accomplishment was non-significant [40]. Higher affective commitment predicted a reduced sense of personal accomplishment, but its predictions on emotional exhaustion and depersonalisation were non-significant [40]. A South Korean police burnout study found that lower organisational commitment was a significant predictor of more burnout [29].

#### 3.4.6. Internal Procedural Justice

Internal procedural justice, that refers to the perceived fairness in the decision-making processes/procedures that police officers receive from their supervisors [64,65], is regarded as a job resource in this review. Among police officers working in the US, unfairness was a significant predictor of more burnout [27,34,60] while an inequitable assignment of cases was also identified as a source of workplace stress [62]. Interestingly, unfairness predicted greater feelings of emotional exhaustion and depersonalisation for male sergeants but not female sergeants [49]. In an Eastern country, perceived unfairness was a significant predictor of elevated feelings of emotional exhaustion and depersonalisation and reduced personal accomplishment for Indian police personnel [57].

#### 3.4.7. Organisational Problems

Mixed results on the prediction of organisational problems were found. In the US, a lack of reward and the absence of a sense of job-related values were significant but weak predictors of greater feelings of emotional exhaustion and depersonalisation [27]. Conflicting information and balancing time between different tasks were identified as sources of workplace stress [62]. A lack of social capital and a larger size of organisation were significant predictors of more burnout [34,35,62]. A lack of social capital means poor and ineffective cooperation between units and trust in work partners. A negative working environment and more sick building syndrome, such as fatigue and shortness of breath in the workplace, predicted a higher level of job stress [41,53]. However, family discussion with co-workers did not predict job stress [41]. Both effort–reward imbalance and overcommitment were significant predictors of greater feelings of cynicism and exhaustion, whereas overcommitment but not effort–reward imbalance predicted lower professional efficacy [55].

Regarding some European countries, among the National police members working in Spain, organisational socialisation (history, language, policies and organisational values) and collective efficacy were non-significant predictors of job stress [37]. In a study conducted on Greek police officers, working out of office was a significant predictor of a higher level of occupational stress [38]. In Scandinavian countries, as for Swedish police personnel, leadership, organisational climate and organisational culture did not predict emotional exhaustion or depersonalisation, except that organisational climate predicted a greater feeling of emotional exhaustion for females [33]. Among Finnish police officers, defective leadership, role conflict and time pressure predicted more burnout [52].

With respect to the countries in Asia, an Indian police burnout study found that lower job involvement was a significant predictor of greater feelings of emotional exhaustion and depersonalisation, but higher job involvement predicted a reduced sense of personal accomplishment [44]. Another Indian police burnout study found that inflexible work hours were a significant predictor of elevated feelings of emotional exhaustion and depersonalisation and reduced personal accomplishment [57]. A South Korean police burnout study found that, although more authoritative culture was a significant predictor of more burnout, poor work condition and lack of collegiate cooperation were non-significant predictors [29].

### 3.5. Operational Predictors

Another source of work-related stress confronting police officers is about the police work content, which is labelled as “operational” factors. A variation of the results relating to operational predictors on police work-related stress was observed. Among those studies conducted in the US, negative exposures such as arresting a violent suspect and exposure to bloody crime scenes were predictive of work-related stress and burnout [30]. Danger, monotony and unpredictability of work were identified as sources of workplace stress [62]. More traumatic events at work, more critical incident strain and greater perceptions of danger were significant predictors of more burnout [34,35,62]. Disciplines were only predictive of depersonalisation for males [49], with male police sergeants who took a less hands-on orientation to discipline perceived a greater feeling of depersonalisation. Regarding some European countries, more threat of violence was predictive of more burnout among police officers working in Finland [52] while more operational stress was predictive of an increased risk of work-related burnout for Turkish National Police members [43]. Finally, a Chinese police burnout study found that more police job stress was a significant predictor of a greater feeling of depersonalisation [59]. More police job stress, indicated by long-time work and training stress, predicted greater feelings of emotional exhaustion and personal accomplishment, respectively. Less job boredom predicted an elevated feeling of personal accomplishment.

### 3.6. Family-Related Predictors

It is acknowledged that some studies in this review found police officers’ work stress and burnout was related to their family circumstances. The predictive power of family-related issues on police work stress and burnout was generally consistent among studies. Instability at home was associated with more burnout for police officers working in the US [34,35], whereas Greek police officers who had less support from family/friends were prone to a high level of occupational stress [38]. Two Indian police studies found that more work–family conflict was a risk factor of a higher level of job stress and burnout [48,57]. Among the four dimensions in work–family conflict, more strain-based conflict, behaviour-based conflict and family-based conflict, but not time-based conflict, were risk factors of a higher level of job stress for Indian police officers [44]. A South Korean police burnout study found that the more work–family conflict, the more the burnout [29].

### 3.7. Community Predictors

In this review, some studies indicated that police work stress and burnout was driven by the community. The prediction of community-related issues on police work stress and burnout varied among studies. In the US, law enforcement officers perceived greater feelings of emotional exhaustion and depersonalisation because of the higher agreement that the public did not understand the meaning of being a police officer [27]. Lack of respect from the community and media members and more criticisms from lawyers and politicians were identified as sources of workplace stress [62]. Among police officers working in the UK, fewer emotional-based public-ascribed trust beliefs in the police emerged as a contributing factor, but reliability and honesty-based public-ascribed trust beliefs in the police did not have an increased risk of workplace stress [46]. For the Spanish National police members, social support was a non-contributing factor of job stress [37]. In Asia, an Indian police burnout study found that community stressors, indicated by political interference and the public’s negative attitude towards the police, had no influence on burnout subtypes [57]. One South Korean police burnout study found that police officers experienced more burnout due to the negative police image [29].

### 3.8. Mediators

Three selected studies examined the mediating effect on burnout and work-related burnout. These studies considered mediators including work–family conflict, resiliency, gender and supervisor support. Among studies conducted in the US, the effect of race on burnout disappeared after adding work–family conflict and resiliency, suggesting that both work–family conflict and resiliency were full mediators [39]. However, gender was a non-significant mediator of burnout [34]. Supervisor support was a non-significant mediator of the relationship between organisational and operational stress and work-related burnout for Turkish National Police members [43].

### 3.9. Moderators

Three selected studies examined the moderating effect on burnout. These studies considered moderators including social support, work support and locus of control personality (internality). Among police officers working in Poland, social support moderated the relationship between job demands and job burnout [58]. When social support was low, increased job demands were associated with a higher level of job burnout. In some Asian countries, an Indian police burnout study found that work support ameliorated the effects of organisational stressors on burnout subtypes [57]. Firstly, a high level of work support offset the impacts of organisational stressors (perceived unfairness and inflexible work hours) on all three burnout subtypes. Secondly, a high level of work support offset the impacts of organisational stressors (role ambiguity and work overload) on reduced personal accomplishment. However, work support did not offset the effects of work–home interface and community stressors on burnout subtypes. A Chinese police burnout study found that locus of control personality (internality) acted as a moderator [54]. With its moderating effect, police stressors (including training stress, long-time work, and job boredom) imposed a greater impact on burnout.

In sum, the aforementioned correlations to police work-related stress can be divided into predictors, mediators and moderators owing to their varying nature of relationship. Six types of predictors are demographic, personal, organisational, operational, family-related and community. Four mediators are work–family conflict, resiliency, gender and supervisor support. Three moderators are social support, work support and locus of control personality (internality).

## 4. Discussion

The current review systematically examined factors affecting police work-related stress directly or indirectly from a multi-level perspective. Focusing on demographics, personal, organisational, operational, family and community predictors, the results enriched the understanding of sources of police work-related stress.

We can understand the sources of police work-related stress at an individual level. Among the demographic factors for police work-related stress, gender is worthy of discussion. A number of studies in the current review yielded mixed results of the gender impact on police work-related stress. Inconsistently, a previous systematic review has found male police officers experienced more stress [16]. This inconsistency can be explained by the dominance of male samples. Regarding personal predictors, a lack of physical exercise was considered to be a stressor in this review. This result is consistent with a previous systematic review [16]. The important role of physical exercise such as yoga or swimming in improving police officers’ well-being is well-researched [66,67]. Personality was also considered to be a personal predictor, with police officers who were optimistic experiencing less job stress. However, a previous systematic review has found that neuroticism and psychoticism were risk factors of stress [16]. It is noteworthy that positive personality traits, i.e., optimism was found in this review, while negative personality traits, i.e., neuroticism and psychoticism, were found in the previous review.

At the organisational level, the core concepts of the JDR model and the JDC model were found applicable to understanding police stress and burnout in more than half of the studies in our review. Police officers who encountered heavy job demands, low job control, a lack of support, and a negative working environment experienced more job stress and burnout. These results are in line with previous systematic reviews [14,16]. Some studies in this review found that police officers who perceived unfairness experienced burnout. This result not only echoes a previous systematic review [14] but also implies the potential role of internal procedural justice as a job resource to reduce police work-related stress. Finally, to the authors’ knowledge, no review conducted found job/workplace satisfaction and organisational commitment as predictors of police work-related stress, highlighting a need for future research to review these results.

At the job level, the microsystem of the ecological model (i.e., the operational aspect of work) and the concept of “job demand” of the JDR model were found to be useful to explain work-related stress among police officers in some studies. Police officers who were exposed to bloody crime scenes, danger and unpredictability of work experienced more work stress and burnout [30,62]. Similarly, a previous systematic review has found that burnout was driven by operational risk factors such as working as an agent and frequent exposure to crimes against children [68]. Consistent with the assumption of the JDR model, operational predictors, as job demands, cause stress and burnout in policing occupations. However, this result contradicts the finding of a previous systematic review that no studies evaluated the relationship between the frequency/severity of critical incident exposures and trauma related disorders, i.e., anxiety and depression [15]. A possible explanation is that work-related stress and burnout were considered in this review, whereas anxiety and depression were considered in the previous review. Regarding search strategy, different search terms and databases were used, so different results were found.

Beyond the personal, organisational, and operational levels, the ecological model has been used to consider occupational stressors at the family and community levels. A previous review applied the ecological model to explain occupational stress among firefighters [19]. This model can be applicable to police officers, since both are first responder occupations and involve highly stressful working conditions. The microsystem of work-related stress in the policing context consists of inadequate support from family. The peri-organisational system influences on police officers include community misunderstanding of being a police officer, community disrespect and more criticisms at the political level.

This review also discussed the mediators (e.g., work–family conflict and resiliency) and moderators (e.g., social support and work support) for police work-related stress. Interventions into the possible mediators and moderators is crucial given some predictors are not amendable, for example race and work demand. Moreover, regional difference was found among the demographic influences on police work-related stress. For instance, educational level and rank were found salient to burnout in the East (i.e., India) but not the West (i.e., the US and European states). It reminds us of a contextual understanding of work stressors.

### 4.1. Practical Implications

Practical implications are offered to reduce police work-related stress. At the personal level, self-positioning in terms of police subculture programs and formal mentoring programs are recommended to deal with the in/out group status [31]. Additionally, two recommendations for maintaining a work–life balance are allowing flexibility of working and arranging reasonable work schedules [59,69]. Responding to the stress of conscience issue, it is important that the police officers are given opportunities to tell supervisors about their troublesome experiences confidentially [70]. At the organisational level, the role of organisational justice and investment of both distributive and procedural justice in the long term are stressed [71]. At the operational level, to minimise the risk of injury, better shift systems designs and improved rotation are suggested [72]. At the family-related level, one suggestion is to create an organisational culture that emphasises the importance of both individual/family life and organisational goals [29]. At the community level, the importance of promoting trustworthy images of the police and community policing (involving engagements and interactions with the community) are highlighted [46].

### 4.2. Limitations and Future Research

Four limitations of this review should be noted when interpreting the results. First, a majority of the studies reviewed are cross-sectional design, which makes it difficult to confirm the causal relationships between variables. For example, although the results indicated that work–family conflicts had an impact on police work-related stress, a reverse causal relationship is possible. The second limitation comes from the geographical variability of study sites that may make research generalisation difficult because the conclusions of one political and cultural context may not be relevant elsewhere. Third, our review relied on studies published in English and excluded those non-English publications. Gender, religious diversity and race issues in police agencies uncovered in non-English articles, for instance, the local journals in Asia, are omitted in our review. This limited our understanding of how far gender, religion and race affected police work-related stress, especially in developing countries where the assurance of quality in the above aspects is not a must. Fourth, the included studies were predominantly based on male samples, so the resulting generalisability to female officers is subject to queries.

For future research, conducting a review of longitudinal studies can help detect the causal and spill-over relationships among work-related stress and stressors, especially the impact of the COVID-19 pandemic over time. Moreover, it would be helpful to narrow the focus of review on special populations such as lesbian, gay, bisexual, and transgender officers, female officers, ethnic minority groups or particular religious groups in the police force. Moreover, evaluating the effectiveness of interventions aiming at reducing police work-related stress is also worth considering.

## 5. Conclusions

This review provided a comprehensive and systematic understanding of sources of police work-related stress, with a focus on demographic, personal, organisational, operational, family-related, and community predictors. Organisational predictors included job demand, job control, job resources, job/workplace satisfaction, organisational commitment, internal procedural justice and organisational problems. This review also clearly provided an understanding of indirect factors affecting police work-related stress by identifying four mediators, work–family conflict, resiliency, gender and supervisor support, and three moderators: social support, work support and locus of control personality (internality). Police work-related stress is a great concern and, therefore, it is important to provide interventions for this occupation.

## Figures and Tables

**Table 1:** Assessment of methodological quality of quantitative studies.

	1. Were the Criteria for Inclusion in the Sample Clearly Defined?	2. Were the Study Subjects and the Setting Described in Detail?	3. Was the Exposure Measured in a Valid and Reliable Way?	4. Were Objective, Standard Criteria Used for Measurement of the Condition?	5. Were Confounding Factors Identified?	6. Were Strategies to Deal With Confounding Factors Stated?	7. Were the Outcomes Measured in a Valid and Reliable Way?	8. Was Appropriate Statistical Analysis Used?
[25]	Yes	Yes	Yes	Yes	Yes	Yes	Yes	Yes
[26]	No	Yes	No	Unclear	No	No	No	Yes
[27]	No	Yes	Yes	Yes	Yes	No	Yes	Yes
[28]	Unclear	Yes	Yes	Yes	Yes	No	Yes	Yes
[29]	No	Yes	Yes	Yes	Yes	Yes	Yes	Yes
[30]	No	Yes	Unclear	Yes	Yes	Yes	Unclear	Yes
[31]	No	Yes	Yes	Yes	Yes	Yes	Yes	Yes
[32]	No	Yes	No	Yes	No	No	No	Yes
[33]	Unclear	No	Yes	Yes	No	No	Yes	Yes
[34]	No	Yes	No	Yes	Yes	Yes	No	Yes
[35]	No	Yes	No	Yes	Yes	Yes	No	Yes
[36]	No	Unclear	Yes	Yes	No	No	Yes	Yes
[37]	No	Yes	Yes	Yes	No	No	Yes	Yes
[38]	No	Yes	Yes	Yes	Yes	Yes	Yes	Yes
[39]	No	Yes	Yes	Yes	Yes	Yes	Yes	Yes
[40]	No	Yes	Yes	Yes	Yes	Yes	Yes	Yes
[41]	No	Yes	Yes	Yes	No	No	Yes	Yes
[42]	No	Yes	Unclear	Yes	Yes	Yes	Unclear	Yes
[43]	No	Yes	Yes	Yes	Yes	Yes	Yes	Yes
[44]	No	Yes	No	Yes	Yes	Yes	No	Yes
[45]	No	Yes	No	Yes	Yes	Yes	No	Yes
[46]	No	No	Yes	Yes	No	No	Yes	Yes
[47]	No	Unclear	No	Yes	Yes	Unclear	No	Yes
[48]	No	Yes	Yes	Yes	Yes	Yes	Yes	Yes
[49]	No	Yes	No	Yes	Yes	Unclear	No	Yes
[50]	No	Yes	No	Yes	No	No	No	Yes
[51]	No	Yes	Unclear	Yes	No	No	Unclear	Yes
[52]	Yes	Yes	Yes	Yes	Yes	Yes	Yes	Yes
[53]	No	Yes	No	Yes	Yes	Yes	No	Yes
[54]	No	Yes	No	Yes	No	No	No	Yes
[55]	Yes	Yes	Yes	Yes	Yes	Yes	Yes	Yes
[56]	No	Yes	No	Yes	No	No	No	Yes
[57]	Unclear	Unclear	Yes	Yes	No	No	Yes	Yes
[58]	No	Yes	Yes	Yes	Yes	Yes	Yes	Yes
[59]	No	No	Yes	Yes	No	No	Yes	Yes
[60]	No	Yes	Yes	Yes	Yes	Yes	Yes	Yes
[61]	No	Yes	No	Yes	No	No	No	Unclear

**Table 2:** Assessment of methodological quality of qualitative study.

Study	1. Is There Congruity between the Stated Philosophical Perspective and the Research Methodology?	2. Is There Congruity between the Research Methodology and the Research Question or Objectives?	3. Is There Congruity between the Research Methodology and the Methods Used to Collect Data?	4. Is There Congruity between the Research Methodology and the Representation and Analysis of Data?	5. Is There Congruity between the Research Methodology and the Interpretation of Results?	6. Is There a Statement Locating the Researcher Culturally or Theoretically?	7. Is the Influence of the Researcher on the Research and Vice Versa, Addressed?	8. Are Participants, and Their Voices, Adequately Represented?	9. Is the Research Ethical according to Current Criteria or, for Recent Studies, and Is There Evidence of Ethical Approval by an Appropriate Body?	10. Do the Conclusions Drawn in the Research Report Flow from the Analysis, or Interpretation of the Data?
[62]	Unclear	Yes	Yes	Yes	Yes	No	No	Yes	Yes	Yes

**Table 3:** Demographic predictors of police work-related stress.

Study	Age	Gender	Education	Marital Status	Family Status	Race	Rank	Tenure	Function
[25]	Yes	No	No	Yes	No	/	No	No	/
[27]	Yes	Yes	/	/	/	Yes	Yes	/	/
[28]	No	No	No	/	/	No	/	/	No
[29]	No	/	No	No	/	/	No	No	/
[30]	/	Mixed	No	No	/	No	/	No	/
[62]	/	/	/	/	/	/	/	/	/
[31]	/	No	No	No	/	Yes	/	Yes	/
[33]	No	/	/	/	/	/	/	/	/
[34]	Yes	No	/	No	No	No	Yes	Yes	/
[35]	Yes	No	/	No	No	No	Yes	No	/
[36]	/	No	/	/	/	Yes	No	Yes	/
[37]	/	/	/	/	/	/	/	/	/
[38]	/	/	/	/	/	/	/	/	/
[39]	No	No	No	/	/	Yes	No	/	No
[40]	No	No	No	/	/	/	No	No	/
[41]	No	No	No	/	/	No	Yes	No	/
[42]	No	No	Yes	/	/	/	Yes	No	/
[43]	/	/	/	/	/	/	/	/	/
[44]	No	No	No	/	/	/	No	No	/
[45]	/	/	/	/	/	/	/	/	/
[46]	/	/	/	/	/	/	/	/	/
[48]	No	/	/	No	/	/	/	No	/
[49]	/	/	No	No	/	Mixed	/	Mixed	/
[52]	/	No	/	/	/	/	/	Yes	Mixed
[53]	Mixed	Mixed	/	No	/	/	/	Mixed	/
[55]	/	/	/	/	/	/	/	/	/
[57]	/	/	/	/	/	/	/	/	/
[58]	Yes	No	/	/	/	/	/	/	/
[59]	/	/	/	/	/	/	/	/	/
[60]	/	Mixed	No	/	/	Yes	/	No	/

## Data Availability

Not applicable.

## Supplementary Materials

The following supporting information can be downloaded at: https://www.mdpi.com/article/10.3390/ijerph20032253/s1, Table S1: Summary of study characteristics and main findings.
