# Supplementary material for: Predictors, Mediators and Moderators of Police Work-Related Stress: A Scoping Review
Source: Int J Environ Res Public Health. 2023 Jan 27;20(3):2253. doi: 10.3390/ijerph20032253 (PMC9916371; doi:10.3390/ijerph20032253)
Supplement: Supplementary file 1 [file ijerph-20-02253-s001.zip › ijerph-2063774-supplementary.pdf]

Table S1. Summary of study characteristics and main findings.

Author details	Study design	Participants'information (N; age; gender; education; marital status; family status; race; rank; tenure; function)	Information of scale/Questions	Mediator/Moderator	Outcome	Location
Wickramasinghe & Wijesinghe (2018)	Quantitative; cross-sectional; self-administrated questionnaire	N=709; 340 aged 20-24 years (48%), 369 aged 41-60 years (52%), M=39.6 years; 591 males (83.4%), 118 females (16.6%); 401 up to General Certificate of Examination (Advanced Level) (56.6%), 308 General Certificate of Examination (Advanced Level) above (43.4%); 580 married (81.8%), 129 unmarried/other (17.2%); 103 no children (14.5%), 606 at least one child (85.5%); no race information; 87 gazetted officers (12.3%), 622 non-gazetted officers (87.7%); 392 up to 20 years (55.3%), 317 > 20 years (44.7%), M=16.8 years; no function information	Questions related to socio-demographic factors, basic employment factors, work environment factors, work pattern factors, work support factors, and work satisfaction factors	/	Burnout subtypes (frenetic, underchallenged, and worn-out)	Sri Lanka
McCarty, Aldirawi, Dewald & Palacios (2019)	Quantitative; cross-sectional; online survey	N=13,146; 2281 aged 18-35 (17.35%), 2118 aged 36-41 (16.11%), 1925 aged 42-45 (14.64%), 2215 aged 46-50 (16.85%), 1913 aged 51-80	Workload<br>Control<br>Rewards<br>Community<br>Fairness	/	Burnout (emotional exhaustion and depersonalization)	US

		(14.55%); 9404 males (71.54%), 1660 females (12.63%); 4921 less than college degree (37.43%), 6331 college degree or above (48.16%); no marital status information; no family status information; 8294 White (63.09%), 911 Black (6.93%), 1002 Latino (7.62%), 578 other (4.4%); 7609 police officer (57.88%), 2279 sergeant (17.34%), 1419 lieutenant or above (10.79%); no tenure information; no function information	The values area of worklife			
Bishopp, Leeper Piquero, Worrall & Piquero (2018)	Quantitative; self-report web-based survey	N=1,400; 18 aged 20-25 (1.3%), 139 aged 26-30 (9.9%), 182 aged 31-35 (13%), 256 aged 36-40 (18.3%), 297 aged 41-45 (21.2%), 255 aged 46-50 (18.2%), 183 aged 51-55 (13.1%), 70 aged ≥ 56 (5%); 84% males, 16% females; 1 = some college, 2 = bachelor's degree, 3 = graduate degree, M=2.6; no marital status information; no family status information; 60% White, 40% non-White; no rank information; no tenure information; 44% patrol, 56 non-patrol	Environmental strain<br>Organizational strain	/	Burnout	US

Yun, Hwang & Lynch (2015)	Quantitative; cross-sectional; questionnaire	N=570; 25-60 years, M=46.1 years; 100% males; high school (1)-graduate degree (7), M=2.83; unmarried (0), married (1), M=.93; no family status information; no race information; policeman (1)-captain (5), M=3.19; 0-36 years, M=19.24 years; no function information	Poor work condition<br>Negative police image<br>Authoritative culture<br>Lack of collegiate cooperation<br>Work-family conflict<br>Lack of supervisor support<br>Organizational commitment	/	Burnout	South Korea
Ivie & Garland (2011)	Quantitative; secondary data	N=600; no age information; military: 217 males (93.9%), 14 females (6.1%), non-military: 302 males (81.8%), 66 females (17.9%); military: 204 less than bachelor's (88.3%), 27 bachelor's and above (11.7%), non-military: 310 less than bachelor's (84%), 54 bachelor's and above (14.6%); military: 140 married (60.6%), 91 not married (39.4%), non-military: 219 married (59.3%), 148 not married (40.1%); no family status information; military: 146 White (63.2%), 83 non-White (35.9%), non-	Negative exposures	/	Work-related stress<br>Burnout	US

		military: 213 White (57.7%), 153 non-White (41.5%); no rank information; non-military: M=9.12 years, military: M=9.81 years; no function information				
Sayed, Sanford & Kerley (2019)	Qualitative; in-depth interviews	N=20; 27-51 years, M=39.7 years; 17 males (85%), 3 females (15%); 60% at least an undergraduate degree, 20 graduate degree; 65% married, 35% unmarried (single/never married, engaged, or divorced); number of children: 0-4, M=1.75; 10 White (50%), 6 Hispanic (30%), 4 Black (20%); no rank information; 5-25 years, total: 310.5 years; no function information	Different types of stressors encountered on jobs (organizational factors, danger, monotony and unpredictability, and community and media scrutiny)	/	Workplace stress	US
Rose & Unnithan (2015)	Quantitative; secondary data	N=1,632; no age information; 87% males, 13% females; 7% high school, 30% some college, 23% associate's degree, 29% bachelor's degree, 6% some graduate school, 4% graduate degree, M=3.10; 72% married, 28% non-married; no family status information; 85% White, 15% non-White; no rank	Out-group status<br>In-group status	/	Job stress	US

		information; 0-30 years, M=12.86 years; no function information					
Backteman-Erlanson, Padyab & Brulin (2013)	Quantitative; cross-sectional; questionnaire	N=856; M=37 years, males: M=40 years, females: M=34 years; 419 males (53%), 437 females (56%); no education information; 670 married/living together (78%), 344 males married/living together (83%), 326 females married/living together (75%); no family status information; no race information; no rank information; M=9 years, males: M=12 years, females: M=6 years; 100% patrol	The General Nordic Questionnaire for and Social Factors at Work<br>Karasek and Theorells demand-control questionnaire<br>The Stress of Conscience Questionnaire	/		Burnout (emotional exhaustion and depersonalization)	Sweden
Gächter, Savage & Torgler (2011a)	Quantitative; secondary data	N=1,104; 20-66 years, M=36.04 years; 943 males (85.73%), 157 females (14.27%); 165 high school (15.08%), 603 some college (55.12%), 285 college (26.05%), 41 graduate school (3.75%); 658 married (59.87%), 88 live-in partner (8.01%), 135 divorced/separated (12.28%), 213 single (19.38%), 5 widowed (0.45%); number of children: 0-7, M=1.18; 355	Social capital<br>Individual fairness<br>Institutional fairness<br>Work-life balance<br>Home index<br>Trauma index	Gender		Burnout	US

		African-American (32.51%), 696 Caucasian (63.74%), 14 Hispanic (1.28%), 27 other (2.47%); 91 officer trainee (8.27%), 601 officer (54.64%), 62 agent (5.64%), 144 detective (13.09%), 143 sergeant (13%), 59 lieutenant/above (5.36%); 0-44 years, M=11.5 years; no function information				
Gächter, Savage & Torgler (2011b)	Quantitative; secondary data	N=1,104; 20-66 years, M=36.04 years; 943 males (85.73%), 157 females (14.27%); college (~26.%), graduate degree (~4%); 658 married (59.87%), 88 live-in partner (8.01%), 135 divorced/separated (12.28%), 213 single (19.38%), 5 widowed (0.45%); number of children: 0-7, M=1.18; 355 African-American (32.51%), 696 Caucasian (63.74%), 14 Hispanic (1.28%), 27 other (2.47%); 91 officer trainee (8.27%), 601 officer (54.64%), 62 agent (5.64%), 144 detective (13.09%), 143 sergeant (13%), 59 lieutenant or above (5.36%); 0-44 years, M=11.5 years; no function information	Social capital<br>Home index<br>Trauma index	/	Burnout	US

Padilla (2020)	Quantitative; cross-sectional; survey	N=147; no age information; males (91%); no education information; no marital status information; no family status information; non-white (55%); patrol (63%); at least 10 years (51%); no function information	Gender<br>Race<br>Rank<br>Tenure	/	Occupational stress	US
Marcos, Pérez-Llantada & Topa (2019)	Quantitative; cross-sectional; online survey	N=123; M=39.77 years; 91.9% males; 30.9% university studies, 22.8% combined their work with some kind of studies; no marital status information; 40.7% had a family member in the National Police; no race information; no rank information; M=171 months; no function information	Organizational socialization (history, language, policies, organizational values)<br>Social support<br>Self-efficacy<br>Collective efficacy<br>Optimism	/	Job stress	Spain
Galanis, Fragkou, Kaitelidou, Kalokairinou & Katsoulas (2019)	Quantitative; cross-sectional; self-completed paper based questionnaire	N=336; M=33.8 years; 256 males (76.2%), 80 females (23.8%); 259 high school graduates (77.1%), 52 university degree (15.5%), 25 MSc/PhD degree (7.4%); 150 married (44.6%), 186 singles/divorced (55.4%); 89 had children < 18 years old (26.5%), 247 did not have children <	Out of office work/Work in office<br>Sleeping hours per day<br>Days of physical exercise per week<br>Self-estimation of family/friends support	/	Occupational stress	Greece

		18 years old (73.5%); no race information; 273 constables (81.2%), 63 sergeants (18.8%); M=12.7 years; no function information				
Griffin & Sun (2018)	Quantitative; cross-sectional; wen-based survey	N=138; 0.73% aged 21-15, 7.97% aged 26-30, 18.8% aged 31-35, 27.5% aged 36-40, 22.5% aged 41-45, 16.7% aged 46-50, 5.8% aged 51-55; 87% males, 13% females; 0.72% high school, 7.25% some college, 15.22% associate degree, 64.49% bachelor degree, 12.32% master degree & above; no marital status information; no family status information; 87% White, 13% non-White; 73.9% corporal or below, 26% supervisor; no tenure information; 54.3% patrol	Demographics	Work-family conflict<br>Resiliency	Burnout	US
Lambert, Qureshi, Frank, Klahm & Smith (2018)	Quantitative; cross-sectional; survey	N=827; 21-57 years, M=36.53 years; 88% males, 12% females; 42% college degree, 58% no college degree; no marital status information; no family status information; no race information; 69% line officer, 31% other position; 0-	Job involvement<br>Job satisfaction<br>Organizational commitment (affective and continuance commitment)	/	Burnout (emotional exhaustion, depersonalization, and reduced sense of personal accomplishment)	India

		30 years, M=2.44 years; no function information				
Tsai, Nolasco & Vaughn (2018)	Quantitative; cross-sectional; secondary data	N=594; 22-62 years, M=36.09 years; 515 males (86.7%), 79 females (13.3%); 188 Bachelor & Higher (31.6%), 406 less than Bachelor (68.4%); no marital status information; no family status information; 478 White (80.5%), 116 Minority (19.5%); 248 supervisor (41.8%), 346 officer (58.2%); 0-30 years, M=12.2 years; no function information	Demographics<br>Negative working environment<br>Counselling support<br>Family discussion with co-workers	/	Job stress	US
Frank, Lambert & Qureshi (2017)	Quantitative; cross-sectional; survey	N=827; 21-57 years, M=36.53 years; 88% males, 12% females; 42% college degree, 58% no college degree; no marital status information; no family status information; no race information; 69% constable, 31% other position; 0-30 years, M=2.44 years; no function information	Job demands (role conflict, role ambiguity, role overload, role underload, dangerousness of job)<br>Job resources (input into decision making, formalization, organizational support, instrumental communication)	/	Work stress	India

Kula (2017)	Quantitative; cross-sectional; self-report survey	N=538; no age information; 92.6% males; 196 two-year college degrees, 238 bachelor degrees; no marital status information; no family status information; no race information; 407 police officers (75.7%); 158 11-15 years; no function information	Organizational Police Stress Questionnaire<br>Operational Police Stress Questionnaire<br>Job Satisfaction Survey<br>Karasek' s Job Content Survey (supervisor support)	Supervisor support	Work-related burnout	Turkey
Lambert, Qureshi, Frank, Keena & Hogan (2017)	Quantitative; cross-sectional; self-report survey	N=827; 21-57 years, M=36.53 years; 88% males, 12% females; 42% college degree, 58% no college degree; no marital status information; no family status information; no race information; 69% line officer, 31% other position; 0-39 years, M=13.82 years; no function information	Work family conflict (strain-based conflict, family-based conflict, behavior-based conflict, time-based conflict)	/	Job stress	India
Padyab, Backteman-Erlanson & Brulin (2016)	Quantitative; cross-sectional; questionnaire	N=856; M=40 years (males), M=34 years (females); 419 males (53%), 437 females (56%); no education information; no marital status information; no family status information; no race information; no rank	Karasek and Theorells questionnaire Job Demand Control-Social Support Model<br>The Stress of Conscience Questionnaire	/	Burnout (emotional exhaustion and depersonalization)	Sweden

		information; no tenure information; 100% patrol				
Rotenberg, Harrison & Reeves (2016)	Quantitative; cross-sectional; online survey	N=183; 22-56 years, M=38 years 11 months; 142 males, 41 females; no education information; no marital status information; no family status information; no race information; no rank information; no tenure information; no function information	Personal trust beliefs in the police<br><br>Public-ascribed trust beliefs in the police	/	Workplace stress	UK
Singh & Nayak (2015)	Quantitative; self-report questionnaire	N=599; 65 below 20 years (10.85%), 98 20-29 years (16.36%), 107 30-39 years (17.86%), 230 40-49 years (38.4%), 99 50 years and above (16.53%); 550 males (91.82%), 49 females (8.2%); 145 under graduate (24.21%), 356 graduate (59.43%), 98 post graduate (16.36%); no marital status information; no family status information; no race information; 100% below inspector level (sub-inspectors, head constables and constables); 90 below 1 year (15.03%), 21 1-5 years (3.51%), 399 6-10 years (66.61%), 89 11 years and	Work-family conflict	/	Job stress	India

		above (14.86%); no function information				
McCarty (2013)	Quantitative; cross-sectional; survey	N=908; no age information; 737 males, 171 females; 536 less than MA (72.7%) (males), 199 MA or above (27%) (males), 108 less than MA (63.2%) (females), 63 MA or above (36.8%) (females); 580 married (78.7%) (males), 154 not married (20.9%) (males), 89 married (52%) (females), 81 not married (47.4%) (females); no family status information; 104 African American (14.1%) (males), 526 White (71.4%) (males), 85 Hispanic (11.5%) (males), 22 Other (3%) (males), 40 African American (23.4%) (females), 110 White (64.3%) (females), 20 Hispanic (11.7%) (females), 1 Other (0.6%) (females); 100% sergeants; 30 6-10 years (4.1%) (males), 199 11-15 years (27%) (males), 211 16-20 years (28.6%) (males), 167 21-25 years (22.7%) (males), 128 26 years or more (17.4%) (males), 12 6-10 years (7%) (females), 29 11-15 years (17%) (females), 61 16-20 years	Views of subordinates<br>Satisfaction with peers and supervisors<br>Work-life conflict<br>Unfairness<br>Discipline	/	Emotional exhaustion<br>Depersonalization	US

		(35.7%) (females), 46 21-25 years (26.9%) (females), 23 26 years or more (13.5%) (females); no function information				
Vuorensyrjä & Mälkiä (2011)	Quantitative; cross-sectional; electronic survey	N=2,821; 26.3% 20-29 years, 43% 30-39 years, 20.9% 40-49 years, 9.8% 50 years; 82.7% males, 17.3% females; no education information; no marital status information; no family status information; no race information; 100% constable; 10.9% < 2 years, 24.3% 2-5 years, 36.3% 6-15 years, 16.6% 16-25 years, 11.9% > 25 years; 20.3% daily crime investigation, 15.8% demanding crime investigation, 63.8% public order and security	Defective leadership<br>Role conflicts<br>Threat of violence<br>Time pressure<br>Shift work	/	Burnout	Finland
Redman, Hamilton, Malloch & Kleymann (2011)	Quantitative; cross-sectional; self-completion paper based questionnaire	N=198; Median= 30-39 years; 66% males, 34% females; no education information; 61% married/living as married, 39% single/divorced/other; no family status information; no race information; 8% inspectors and above, 20% sergeants, 53% constables, 18% others; Median= 10 years	Hours spent in the workplace building<br>Positive affectivity<br>Sick building syndrome	/	Job stress<br>Burnout	US

		or more; no function information				
Violanti et al. (2018)	Quantitative; cross-sectional; self-report survey	N=200; M=46.2 years; 143 males (72%), 57 females (28%); 13 high school/GED (6.5%), 103 college < 4 years (51.5%), 84 college 4+ years (42%); 20 single (10.1%), 137 married (68.8%), 42 divorced (21.1%); no family status information; 162 Caucasian (81%), 38 African American (19%); 110 police officer (55%), 39 sergeant/lieutenant (19.5%), 51 captain/detective (25.5%); 9 0-9 years (4.5%), 45 10-14 years (22.5%), 68 15-19 years (34%), 78 20+ years (39%); no function information	Effort-reward imbalance Overcommitment	/	Burnout (cynicism, exhaustion, and professional efficacy)	US
Kumar & Kamalanabhan (2017)	Quantitative; survey	N=491; aged 31-39 years (43%); males (81.7%); no education information; no marital status information; no family status information; no race information; sub-inspectors (54.7%); 2 years; no function information	Organizational stressors (perceived unfairness, inflexible work hours, work overload and role ambiguity)<br><br>Community stressors (publics	Work support	Burnout	India

			negative attitude toward police and political interference)<br><br>Work–home interface (work-family conflict and family–work conflict)			
Smoktunowicz et al. (2015)	Quantitative; cross-sectional; secondary data	N=607; 21-61 years, M=36.64 years; 483 males (80%), 124 females (20%); no education information; no marital status information; no family status information; no race information; no rank information; 1-36 years, M=12.83 years; no function information	Job demands<br>Job control<br>Social support	Social support	Job burnout	Poland
Wang, Zheng, Hu & Zheng (2014)	Quantitative; survey	N=521; 192 aged 40-50 (36.9%), 162 aged 30-40 (31.1%); 392 males (75.2%); no education information; no marital status information; no family status information; no race information; no rank information; no tenure information; 192 public security (36.4%), 69 criminal	Police job stress	Locus of control personality (internality)	Job burnout	China

		investigation (13.1%), 51 office (9.7%)				
McCarty & Skogan (2013)	Quantitative; online questionnaire	N=2,564; no age information; 1871 males, 663 females; 1152 less than Bachelor's degree, 1298 Bachelor's degree or higher; no marital status information; no family status information; 365 African American, 1508 White, 422 Hispanic, 197 Others; no rank information; M=12.93 years (civilian), M=17.17 years (sworn); no function information	Job related (perceptions of danger, work-life conflict, shift assignment work)<br>Organizational (social support from coworkers and supervisors, perceived unfairness of the organization, size of the organization)	/	Burnout	US
